# Kidney sales by migrants: experiences, knowledge, and responses of medical professionals across the euro-mediterranean border

**DOI:** 10.3389/ti.2026.16783

**Published:** 2026-08-24

**Authors:** Zanna Ramaekers, Richard Staring, Dennis A. Hesselink, Frederike Ambagtsheer

**Affiliations:** 1 Section of Nephrology and Transplantation, Department of Internal Medicine, Erasmus MC Transplant Institute, University Medical Center Rotterdam, Rotterdam, Netherlands; 2 Department of Law, Society, and Crime, Erasmus School of Law, Erasmus University Rotterdam, Rotterdam, Netherlands; 3 Bureau Beke, Arnhem, Netherlands

**Keywords:** medical professionals, migrants, organ traffickings, organ transplantations, transplant professionals

## Abstract

Reports indicate that migrants are selling their kidneys, yet medical professionals’ experiences with this are unexplored. We conducted a qualitative study with 45 professionals across the Euro-Mediterranean border (The Netherlands, Bulgaria, Türkiye, Italy, and Greece) working in transplantation and migration. We examined their experiences, knowledge, and responses to migrants’ kidney sales and explored whether the issue is rarely encountered, insufficiently recognized, or disclosed. The study includes 28 transplant professionals (TPs) and 17 medical professionals working in migration (MMPs). Except for two TPs volunteering in migration settings, none reported direct encounters with migrant kidney selling. In contrast, five MMPs observed nephrectomy scars or heard testimonies. Awareness was largely limited to those with direct experience, and knowledge on modus operandi was absent. Both groups expressed uncertainty about detecting and reporting suspected cases. TPs tended to distance themselves and, like MMPs, acknowledged that limited awareness may reduce attentiveness. We conclude that migrant kidney selling remains largely hidden in medical practice, not because it is absent, but because it is insufficiently recognized and rarely disclosed. To address this, we recommend stronger collaboration through conferences, multidisciplinary training, and education on organ trade, as well as expanding reporting mechanisms to include migrant kidney sellers.

## Graphical Abstract

**Figure:**
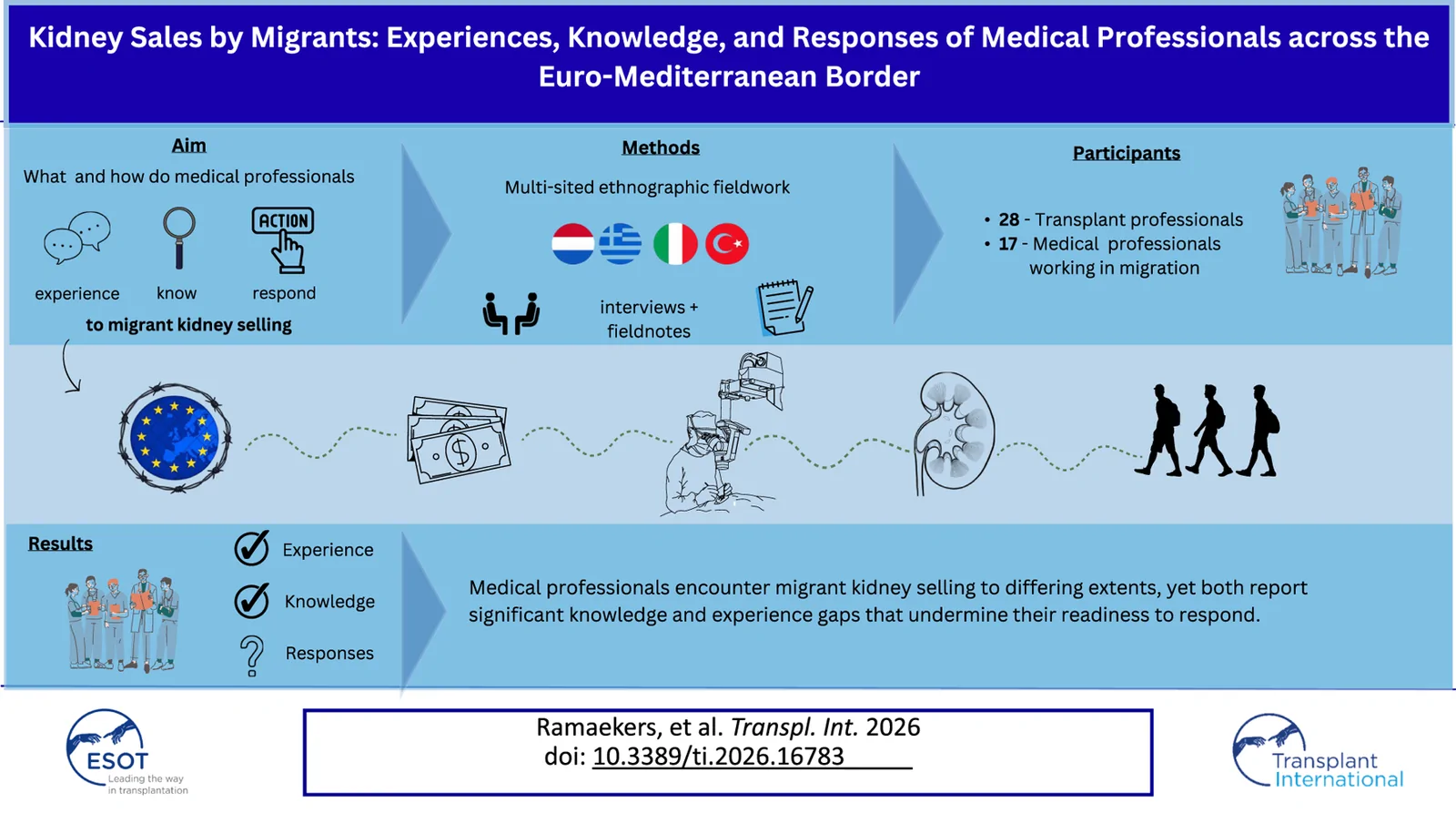

## Introduction

A growing number of reports indicate that migrants are selling their kidneys during their journeys [1–3]. As anti-migration laws and policies are becoming ever-more stringent, human smuggling and trafficking economies expand and intersect, with the trade in living donor kidneys representing one of these new convergences [2, 3]. Although the harms associated with kidney sales are well documented [4–6], studies on kidney sales by migrants remain scarce [7]. Existing evidence largely comes from documented cases in Egypt, where migrants have had their kidneys forcibly removed or voluntarily sold [8, 9]. Kidneys are typically sold to finance their journey to Europe, pay off debts to smugglers, or to survive while they are unable to continue their migration journey. Although the organ trade has been estimated to rank among the most lucrative transnational crimes [10], it remains largely undetected [11, 12]. The strategic targeting of undocumented migrants further reinforces the invisibility of the kidney trade through processes of double criminalization: many migrants who sell their kidney, lack valid documents [8]. This dual positioning limits any legal protection and thus further discourages them from reporting abuse [8]. As the structural drivers of migrant kidney sales intensify (organ scarcity and increasing exclusionary migration policies), this problem is likely to persist or even expand.

Kidney sales by migrants necessarily involve medical professionals. While some studies document perspectives of transplant professionals towards organ trade, in particular to patients’ organ purchases [13, 14], and the importance of their role in curbing the issue [15], there is limited research on the encounters and responses of medical professionals with kidney sales by migrants [2]. We conducted a qualitative study with medical professionals working in transplantation and migration in The Netherlands, Bulgaria, Türkiye, Italy, and Greece to examine their experiences with, knowledge of, and responses to migrants’ kidney sales. Drawing on these insights, this study explores if and how the issue is encountered and responded to. It also identifies factors that may hinder the identification or reporting of such cases, helping to explain its marginal position in medical discourse and underscoring the need for clearer reporting pathways within healthcare systems and for migrant populations.

## Materials and methods

### Participant selection

Participants were interviewed as part of a larger multi-sited ethnographic fieldwork study, ‘Organs as a Gateway to Europe’ [16], that ran from 2021 until 2026 across the Euro-Mediterranean border. This study focused on migrants in The Netherlands, Bulgaria, Türkiye, Italy and Greece who sold, or contemplated selling, their kidneys. The Ethics Review Committee of the Erasmus School of Law of the Erasmus University Rotterdam provided ethics approval [ETH2122-006], and the study received funding from the Dutch Research Council (VI.Veni/201R.010, 2021-2026).

Countries were selected based on their geographic position along the Eastern Mediterranean migration route. This constitutes a key migration corridor into Europe [17] and has not been previously studied in the context of migrant kidney sales. We did not conduct research in countries across the Central Mediterranean route, because this region has previously been explored by Columb [2, 3, 8, 9]. In addition, due to time and funding restrictions, it was not feasible for the research team to include more field sites.

In each of the countries, medical professionals were purposively approached who worked in the field of migration or in the field of transplantation, first, because knowledge about their experiences with migrants’ kidney sales is scarce. Second, we anticipated that due to the nature of their profession, they would be able to provide relevant insights into our research aims.

In lieu of the explorative, qualitative nature of our study, participants were approached using a combination of opportunistic and convenience sampling techniques. Initially, transplant professionals (TPs) who were known to the authors were emailed and invited for an interview. These included transplant professionals known through professional affiliations and networks such as Erasmus MC, the European Society for Organ Transplantation (ESOT), the European Platform on Ethical, Legal and Psychosocial aspects of Transplantation (ELPAT) and the Dutch Transplantation Society. These professionals further nominated prospective respondents, whom they believed could share experience, knowledge, and perspectives about migrants’ kidney sales.

The majority of health professionals working in migration (MMPs) were not previously known to the authors. Through volunteer ethnography in the various field sites, connections were formed with professionals working for non-governmental organizations who provided care and support for migrants. These participants were approached via face-to-face introductions from interlocutors, independently visiting their offices, or via email.

Our sampling strategy resulted in 45 interviews, 28 with TPs and 17 with MMPs. TPs included transplant surgeons, nephrologists, nurse practitioners, transplant coordinators, social workers, and psychologists. MMPs included general practitioners, a urologist, a gynecologist, a pulmonologist, and nurses working or volunteering for health clinics in asylum centers, mobile health clinics, medical humanitarian agencies, and hospitals. The participant characteristics are presented in Table 1.

**TABLE 1:** Participant characteristics.

Transplant professionals				
Country	Position	Years of experience Junior >5 years Mid-level >10 years Senior >20 years	Sex (F/M)	Work/volunteer place
Greece	Transplant surgeon	Senior	M	Academic and public hospital
Greece	Transplant surgeon	Senior	M	Academic public hospital
Greece	Transplant surgeon	Intermediary	M	Academic public hospital
Greece	Transplant surgeon	Junior	F	Academic public hospital
Greece	Nephrologist	Senior	M	Academic public hospital
Italy	Transplant surgeon	Intermediary	F	Academic public-private hospital^1^
Italy	Transplant surgeon	Intermediary	M	Academic public hospital
Italy	Patient coordinator	Senior	M	Academic public-private hospital
The Netherlands	Nephrologist	Senior	F	Academic public hospital
The Netherlands	Nephrologist	Senior	F	Academic public hospital
The Netherlands	Nephrologist	Senior	F	Academic public hospital
The Netherlands	Psychologist	Senior	F	Academic public hospital
The Netherlands	Nurse practitioner	Intermediary	F	Academic public hospital
The Netherlands	Nurse practitioner	Senior	F	Academic public hospital
The Netherlands	Social worker	Senior	M	Academic public hospital
The Netherlands	Transplant surgeon	Senior	F	Academic public hospital
The Netherlands	Transplant surgeon	Senior	M	Academic public hospital
The Netherlands	Transplant surgeon	Senior	M	Academic public hospital
The Netherlands	Transplant surgeon	Senior	F	Academic public hospital
Türkiye	Nurse practitioner	Senior	F	Academic public hospital
Türkiye	Transplant coordinator	Senior	F	Academic public hospital
Türkiye	Transplant coordinator	Intermediary	F	Public-private hospital
Türkiye	Transplant surgeon	Senior	M	Public- private hospital
Türkiye	Transplant surgeon	Senior	M	Public-private hospital
Türkiye	Transplant surgeon	Senior	M	Public-private hospital
Türkiye	Transplant surgeon	Senior	M	Academic public hospital
Türkiye	Transplant surgeon	Senior	M	Academic public hospital
Türkiye	Transplant surgeon	Intermediary	M	Private hospital
Medical professionals working in migration				
Greece/Türkiye	General practitioner	Senior	M	International humanitarian organization
Italy	General practitioner	Senior	M	International humanitarian organization
Italy	General practitioner	Intermediary	F	International humanitarian organization
Italy	Nurse	Intermediary	M	Mobile clinic of an international humanitarian organization
Bulgaria	Nurse	Intermediary	F	Activist organization
The Netherlands	General practitioner	Senior	F	Asylum seekers’ centre
The Netherlands	General practitioner	Intermediary	M	Asylum seekers’ centre
The Netherlands	General practitioner	Intermediary	M	Asylum seekers’ centre
The Netherlands	Nurse	Junior	F	Asylum seekers’ centre
The Netherlands	Nurse	Senior	F	Asylum seekers’ centre
The Netherlands	Nephrologist	Senior	M	Public hospital
Türkiye	Gynecologist	Senior	M	Private healthcare clinic
Türkiye	Urologist	Senior	M	Private healthcare clinic
Türkiye	General practitioner	Senior	M	Private healthcare clinic
Türkiye	General practitioner	Senior	M	National medical nongovernmental organization
Türkiye	Pulmonologist	Senior	M	Public hospital
Türkiye	Psychologist	Intermediary	M	National medical nongovernmental organization

1 The hospital is integrated into the regional public health network and regulated by the Ministry of Health but managed through a private international nonprofit healthcare system.

### Data collection

ZR conducted the majority of the interviews while FA conducted a minority between March 2023 and February 2026. Data saturation was reached among both groups, with themes regularly re-emerging, before all interviews were completed. All participants verbally consented to the interviews, 23 of which were audio-recorded and transcribed. Given the sensitivity of the research topic, the other 22 medical professionals did not want to be recorded. In these cases, detailed notes were taken instead and included in the coding and analysis. Recorded interviews were semi-structured following a topic list pertaining to professional background, local medical/transplantation context, migrant patients/donors, migrant kidney selling, and organ trade. Non-recorded interviews followed the same topic list but were discussed with an open-ended interview approach. The detailed topic list is included as Supplementary Appendix 1.

Apart from two online interviews with MMPs, all interviews were conducted face-to-face. Interviews took place at transplant conference venues, inside respondents’ offices, or in public areas selected by the respondents. The interviews lasted between 30–60 min and were performed in English, French, or Dutch.^1^

To ensure data protection and participant safety, the research team adopted specific security measures. All respondents were informed that their names would be pseudonymized. Interviews were recorded using double authenticator work phones and audio-records were immediately uploaded to a protected digital environment of Erasmus MC and thereafter, immediately deleted from the phone recording devices. Transcripts were pseudonymized and fieldnotes were anonymized to further enhance participant confidentiality. An encrypted pseudonym key file was stored separately from the dataset.

### Analysis

The transcripts were imported into NVivo software [Nvivo 15.4 (c) Version 3.0] for coding qualitative data. ZR inductively coded all transcripts into thematic concepts. Two coding structures (one for each group), across all countries, were generated. For each group, similar codes were grouped into themes. Interpretations of themes, conceptual relationships, and similarities and differences between and among the two groups were discussed during multiple, team-based analytic discussions to ensure that the full breadth of data was captured. The themes were organized around the relevant domains of “experience,” “knowledge,” and “response.” “Experience” was defined as “hearing testimonies from migrants, seeing suspicious scars on migrant bodies, or receiving inquiries from migrants, and for transplant professional, having a (potential) donor who was an asylum seekers, refugee, or undocumented migrant.” “Knowledge” included “being aware of migrant kidney selling, the speculations TP and MMPs had about migrant patients/donors as well as the intersection of migration and transplantation, stories they heard about migrant kidney selling, and the limitations of their knowledge on migrant kidney selling.” “Response” was divided into “attitudes” and “reactions.” Attitudes were understood as their behaviour or opinion towards migrant kidney selling. “Reactions” included their “actions, or lack thereof, in understanding or reporting migrant kidney selling.”

The multidisciplinary background of the research team (i.e., anthropology, sociology, criminology and nephrology) ensured that various interpretations and perspectives were taken into account, encouraged reflexive considerations and reduced risk of discipline-specific biases. Reliability of findings was further enhanced by triangulating data from different types of respondents from different geographic locations and by involving multiple researchers in data collection, analysis and reporting.

## Results

Results reveal that MMPs and TPs in countries across the Eastern Mediterranean migration route have incidentally treated migrants who have sold a kidney. For those who have not treated migrant kidney sellers, the issue is largely a matter of hearsay. In what follows, we elucidate our findings in more detail, focusing on how the experience, knowledge, and responses of TPs and MMPs give insight into how the issue is encountered and responded to.

### Direct experiences with migrants’ kidney sales and “hear say”

Of all respondents, mostly MMPs reported direct experiences with migrants who had sold a kidney. In contrast, only very few TPs had experiences with this issue. One nephrologist and one transplant surgeon reported direct encounters with migrants who had sold a kidney. They described observing nephrectomy scars and, upon inquiry, receiving either an evasive response (interpreted by the professional as indirectly suggestive of organ trade), or an explicit admission that the kidney had been sold. Importantly, their reported experiences were not based on their work within transplant settings, but on their involvement as medical volunteers in the migration field. TPs who exclusively work in transplant settings report more speculative accounts. For example, one TP shared an instance where a medical visa was arranged for a donor who traveled from abroad to donate to a recipient. However, the donor never showed up for the scheduled surgery. Another TP shared receiving anonymous kidney offers which he assumed to be from migrants, because those offering were of African origin.

Overall, TPs reported very limited interactions with migrants as donors, more commonly treating migrants as renal patients. Most TPs described their experiences with organ trade through assumptions relating to transplant tourism, i.e., patients traveling abroad for dubious kidney transplantations.

*“This patient could not wait, so she, I do not know how, but she selected this option* [going to India and getting a new kidney] *in order to, I think save her life probably. But generally, apart from documentaries or something, I do not have experience with that, but I do believe that poverty and medical needs, leads you to this black market*” [TP, Greece, 2024]

Contrastingly, five MMPs spoke of seeing nephrectomy scars and, similar to the two TPs, shared receiving defensive responses upon their enquiries, or confirmation that they had sold their kidney. The timing of these encounters varied: one participant experienced it a year ago, another 3 years ago, two during the 2015 ‘migration crisis’, and one MMP did not provide an answer. Two others shared experiences of migrants consulting them for information about transplantation procedures, revealing they were considering selling their kidney. One MMP experienced this 5 years ago and the other did not recall years but described it as “*the man it was a while ago, but the woman it is recent.*”

*“Of course, there are patients who came, some came and said they wanted to cross in the summer, so they came to sell their kidney to pay the debt, or they came with someone who has already sold their kidney, there are some wherein I cannot go into detail, but they are there, men and women.''* [MMP, Türkiye, 2025]

### Knowledge and awareness about migrants’ kidney sales

Except for the two TPs who worked as volunteers in the migration field and the transplant surgeon who received anonymous kidney offers assumingly from migrants, all other TPs were unaware of migrant kidney selling. Instead, TPs reported knowledge about organ trade generally based on cases of transplant tourism, rumors, media reports, and conference presentations. Their awareness thus largely stems from second-hand stories.

*“People, or doctors, approaching or a system approaching poor people and giving them some money in exchange for their kidneys. […] There are stories of whole villages with people who have one kidney because they sell their kidney. I have heard such stories yes, but I cannot confirm them.”* [TP, Greece, 2023]

Among MMPs, awareness of migrant kidney selling was primarily limited to those with direct experiences, and those who had learned about such cases through colleagues. However, some MMPs were aware of the limitations of their consultations which constrained their awareness of this issue. They explained that unless a migrant travels through a tuberculosis-risk region or requests a physical examination, their bodies are rarely examined, leaving potential scars unnoticed. *‘'If the resident* [asylum seeker] *does not say anything, we’ll never find out. Or you really have to be lucky that the same person comes to us with stomach pain. Then you look at them and ask, ‘What’s that scar?’ But the chance is small, because during the check you’re not facing their back. And that cut is over there, on the flank. Yeah, it will not always be noticeable to me.”* [MMP, The Netherlands, 2025]

MMPs added that without prior awareness of migrant kidney selling, nephrectomy scars do not necessarily arouse curiosity, given the multitude of scars routinely observed on migrant bodies. Possible identification of signs of migrant kidney selling thus largely relies on the proactiveness of MMPs and the willingness and courage of migrants to share their experiences. MMPs noted however, that migrants will be unlikely to share their stories, especially if they are under the unjustified belief that revealing a punishable activity may hinder their asylum application.

### Responses to migrants’ kidney sales

When asked how they (would) respond(ed) to migrants who sold, or wanted to sell a kidney, most MMPs and TPs expressed uncertainty on when, where and how to report such cases. None of the instances shared by MMPs and TPs were reported to authorities. Both TPs and MMPs stated that they (would) treat(ed) the migrant and that they (would) discuss(ed) the case with their colleagues.

Given their limited experience and knowledge of migrants’ kidney sales, most TPs spoke about organ trade in more general terms. They explained that they find it challenging to make organ trade suspicions concrete. Most of their experience concern speculations and most of their knowledge stems from second-hand accounts. Some TPs shared that once a donor is approved on paper, few critical questions follow, despite having suspicions.

“*There are these big systems in place to check these things but of course you can cheat the system, but I think the fact you can cheat the system is ignored because we can perform a transplant, and that is a priority. If on paper it is approved, then there are little critical questions asked, which is concerning to me as well''* [TP, Italy, 2024]

Others expressed a need for guidance and protection in handling suspicions of organ trade:

*“I believe we should have an established protocol that we would be protected, because imagine if I am mistaken, imagine if you have a sentimental donor that values his or her decision and I say ‘no you took money’ then first of all I bring down all the system of transplantation because I insult him. Second, I am wrong.”* [TP, Greece, 2023]

Most TPs who recounted organ trade suspicions, including those with direct experience, quickly added ''*having forgotten the details*.” Most had shared the case within their multidisciplinary teams. Others explained being cautious about sharing their thoughts about organ trade suspicions with patients and colleagues, fearing potential reputational harm. Some TPs kept cases to themselves, regarding it as patient confidentiality. A TP mentioned:

“*I also have very little experience with it myself, so I hope, in any case, that it’s true that it really occurs so rarely. It’s quite possible that we sometimes have blinders on. The tricky part is that you often do not realize it yourself. Yes. So, I think it would be good if people with a different perspective on things worked together with us.*” [TP, The Netherlands, 2025]

While few TPs accepted that organ trade could occur in Europe, including in their own transplant facilities, most others assumed that organ trade could not occur in their facilities, due to thorough donor screening, ethics committees, state oversight, and the small, interconnected transplant community. Yet, again, they acknowledged that their lack of knowledge and awareness may unwittingly narrow their perception and attentiveness to organ trade.

*“We have all heard stories, dreadful stories, I think I can speak for the European part, for the ESOT [European Transplantation Society]. I do not think it concerns them a lot, no. Maybe it would be a good idea to have more resources pointed to countries but maybe not in Europe. We should monitor it maybe better*'' [TP, Greece, 2023]

## Discussion

Existing studies mainly elucidate experiences and responses of transplant professionals towards transplant tourism [13–15, 18]. Scholarship on the exploitation of migrants mainly centers on labour and sexual exploitation, largely overlooking (health) vulnerabilities relating to organ trade and trafficking [19, 20]. Our study reveals that medical professionals working in transplantation and migration in countries across the Euro-Mediterranean border, also encounter migrant kidney sales. Given the explorative nature of our study and the lack of systematic reporting, the identified cases in our research likely do not represent the problem’s true scale. Other limitations include the restricted geographic scope due to time and resource constraints, and the uneven distribution of participants across countries, with a greater number of Dutch and Turkish participants reflecting stronger professional networks in the Netherlands and a longer period of fieldwork in Türkiye. Nevertheless, our findings raise implications for both medical communities.

First, MMPs report more experience, knowledge and awareness than TPs. It is noteworthy that the only two TPs who reported experience and knowledge of migrant kidney sales, worked as volunteers in the migration field, alongside their jobs as transplant professionals. This suggests that social engagement by transplant professionals could strengthen their experience, knowledge, and awareness of this neglected issue. All other TPs, through the nature of their professions, reported limited to no direct experiences with refugees, asylum seekers, or undocumented migrants as living kidney donors and/or sellers. Access to living kidney donation is highly regulated through medical, administrative and legal criteria, which generally excludes individuals lacking a residence status. As such, refugees, asylum seekers, and undocumented migrants who (may) have sold a kidney, remain largely invisible to TPs. Moreover, donors usually receive only a fixed period of medical follow-up, and lifelong donor care is not provided in most European countries which limits opportunities for transplant professionals to notice them [21]. As noted, TPs more often encounter migrants as patients requiring renal care and incidentally treat patients who travel abroad for organ transplants. Their experiences, knowledge and awareness of organ trade-related problems are thus shaped by the patient populations they treat.

The nature of MMPs’ professions and their proximity to migrants allows them to more readily identify migrants’ kidney sales than TPs. However, restrictive migration policies have reduced the presence of medical humanitarian organizations, limiting MMPs’ opportunities to observe the issue [22]. The lack of tailored questioning, the absence of physical examinations and migrants’ reluctance to share their accounts during screening in asylum centers, further hampers awareness and knowledge of the issue.

Despite their differing professions and experiences, both TPs and MMPs report gaps in knowledge and awareness that challenge their readiness to identify or respond to migrant kidney selling. While many of these gaps are shaped by structural factors, they are also, to some extent, sustained by limited attention given to signs of migrant kidney selling, and by the fragmentation of information about this issue. Compared to MMPs, we found that TPs, despite their direct and indirect experiences with organ trade, were more inclined to distance organ trade-related problems from their profession. They claimed that the activity occurs in other geographic regions (namely, the global south), in private clinics, or among foreigners attempting to circumvent donor screening, thereby reinforcing popular depictions of the trade as a concern that does not take place under European jurisdiction. This suggests the need for knowledge–and awareness-raising amongst TPs about organ trade in Europe.

Previous studies have pointed out limitations in documenting, tracking, and responding to organ trade, as well as elucidating cultures of silence and secrecy, or passive responses among transplant professionals [16, 17, 19, 22]. Yet, professional transplant groups and other authors have stated that transplant professionals are well positioned to assume a monitoring role in reporting organ trade cases [23]. Under auspices of the Council of Europe, various legal and regulatory frameworks have been adopted urging governments to collect data on illicit transplant activities and on patients who travel abroad for transplantations [23–27]. Under the Council of Europe’s Network of National Focal Points on Travel for Transplantation (NETTA) [28] countries are establishing mandatory reporting tools for transplant professionals, including the United Kingdom, Spain, Netherlands, Hungary and Portugal. However, many of these codes focus on collecting information about patients travelling abroad for transplantation, and not on migrants’ kidney sales or those that facilitate illegal kidney transplant operations [28–30]. Previously, the key elements for a reporting code on disclosure of information of organ trafficking networks have been outlined [31, 32]. These codes could be strengthened by a) including information about (migrant) kidney sellers and b) encouraging the sharing of this information between authorities to disrupt criminal networks and initiate protection mechanisms for migrant kidney sellers. These codes can further help to estimate the scale of this neglected issue.

In addition, our findings demonstrate the importance of developing partnerships between professionals working in transplantation and migration. These multidisciplinary partnerships can result in knowledge transfer, training and safeguarding measures for migrant kidney sellers, In addition, these partnerships can strengthen awareness and expertise on how to identify and address the problem, including how reporting can be improved. This can be achieved, for instance, by uniting professionals from both fields in international conferences and symposia and fostering multidisciplinary collaborations. Finally, results suggest that both MMPs and TPs would benefit from education and training on the possible criminal context of organ trade more generally (including the fact that the trade does not only occur in the global south, but also in European transplant centers), on identifying possible migrant kidney sellers, on recognizing scars indicating kidney removal, and on probing this delicate issue amongst migrants during screening and care.

## Data Availability

Raw data will be provided in anonymized form and only in so far that it does not harm or incriminate the persons and institutions involved in this study.

## Footnotes

1 ZR is a native French, English and Dutch speaker, and translated relevant transcripts to English.
