# Supplementary material for: Kidney sales by migrants: experiences, knowledge, and responses of medical professionals across the euro-mediterranean border
Source: Transpl Int. 2026 Aug 24;39:16783. doi: 10.3389/ti.2026.16783 (PMC13575741; doi:10.3389/ti.2026.16783)
Supplement: Supplementary file 2 [file Supplementaryfile2.docx]

**Appendix**

Interview topic guides

Transplant professionals

1. Professional Background:

- Can you describe your role and main responsibilities?
- How long have you worked in this position?

2. Local Context

- How many transplants are performed annually at your hospital?
- How many are from living vs. deceased donors?
- How would you describe the national transplantation culture?
- Public attitudes toward donation
- Trust in healthcare

3. Donation Process and Safeguards

- Who typically becomes a donor?
- Can you describe the donor screening process?
- Key steps and professionals involved?
- What safeguards ensure donation is voluntary?
- How do you assess informed consent?

4. Pressure and Ethical Concerns donors

- Have you ever encountered a donor who seemed pressured?
- What kind of pressure did they face? Family, relationship, financial...
- How was it handled?
- How confident do you feel in the donor screening process to identify coercion?
- Have patients asked about buying an organ?
- Have you encountered patients who traveled abroad for transplantation?

      5. Organ Trade

- What has been your exposure, if any, to the issue of organ trade?
- Heard stories, had suspicions, had cases?
- Patients traveling abroad and returning with dubious new kidney?
- Offers to sell kidney?
- How vulnerable do you think European transplant systems are to organ trade?
- Is organ trade a concern in the transplant community?
- If so, what do they do about it?

6. Organ trade and migration

- What, if any, has been your experience with irregular migrants, asylum seekers, refugees wanting to be a donors/recipient?
- Can they become donors/recipients?
- Are their screenings different than others?
- Have you heard stories, reports about, or cared for a migrant (who considered/sold their kidney? )
- Do you think such cases would be visible within the transplant system?
- What is your perspective on this issue?
- What do you think, if any, is the responsibility of the transplant community to address this issue?
- Do you think current systems are doing enough to prevent organ trade and trafficking? Why or why not?
- Where do you see gaps, if any, in prevention or oversight?

7. Wrap up

- What role should the transplant community play in addressing organ trade (in context of migration)?

Medical Professionals in working in migration

1. Medical professionals working in migration Background:
- What is your function?
- How long have you been working in this positions?

2. General Screening Procedures
- Could you explain to me what the standard medical screening for migrant patient entails?
- Are these screenings mandatory?
- Are there differences in screening based on age, gender, nationality, migration route?
- How is further medical follow-up organized after the initial screening?
- How much time is typically spent on a medical screening per person?
- Are there shortages in resources or personnel that affect the quality of the medical screening?

3. Trust

- Are migrants willing to come for medical screenings/physical examinations?
- Do migrants seem comfortable when they talk with you?
- Do migrants usually return for follow-up visits when you ask them to?

4. Specific Health Concerns

- Are there specific conditions or health problems that occur more frequently among asylum seekers?
- Do asylum seekers have access to specialist care?

5. Suspicions

- During the screening, are signs of human trafficking, sexual violence, or other forms of abuse detected?
- If so, did you see any scars pointing towards a renal transplantation?  (nephrectomy scars)
- Is there a protocol to ask questions about scars?
- Do you ask questions if you see suspicious scars?

6. Organ trade and migration

- What has been your exposure, if any, to the issue of organ trade?
- Heard stories, had suspicions, had cases?
- Did you ever have migrants requesting to sell a kidney?
- Did you ever care for migrants who sold a kidney?

7. Response

- What did you/ would you do if becoming aware of a migrant who sold his kidney?
- Is there a protocol to report such cases?

8. Warp up

- Is the current medical screening in the asylum center thorough enough to identify signs of the organ trade?
- Do healthcare staff receive adequate training to recognize and safely respond to possible indicators of organ-trafficking–related harm?
- What improvements could be made to strengthen the system’s ability to detect and safeguard migrants who may have experienced medical exploitation?
